# Vitamin D testing in pharmacies: Results of a federal screening campaign

**DOI:** 10.1016/j.rcsop.2025.100596

**Published:** 2025-03-25

**Authors:** Olaf Rose, Stefanie Eppacher, Johanna Pachmayr, Stephanie Clemens

**Affiliations:** a Institute of Pharmacy, Pharmaceutical Biology and Clinical Pharmacy, Paracelsus Medical University Salzburg, Salzburg, Austria; b Center of Public Health and Health Services Research, Paracelsus Medical University, Salzburg, Austria

**Keywords:** Screening, Prevention, Vitamin D, Community pharmacy, Professional development, Clinical pharmacy services

## Abstract

**Introduction:**

The convenient accessibility of pharmacies positions them as optimal venues for screening initiatives. There is growing public concern regarding vitamin D levels, particularly during the winter months in middle-and northern latitudes. This study aimed to assess vitamin D levels in early spring and to evaluate the feasibility of implementing a large-scale screening campaign within pharmacies.

**Methods:**

This investigation was structured as a cross-sectional multicenter survey conducted in a routine care setting. Data collection was performed in a fully anonymized manner. An automatic fluorescent immunoassay system was utilized for point-of-care-testing, and both patient and provider questionnaires were developed. Quantitative data were analyzed descriptively. Both parametric- and nonparametric statistical tests were performed to analyze the data, ensuring robust results across different assumptions. A resulting maturity matrix for implementation was conceptualized.

**Results:**

Over a two-week campaign, 62 community pharmacies conducted a total of 2770 vitamin D tests. All participants completed the questionnaire, and 45 pharmacists participated in a retrospective survey. Vitamin D deficiency was identified in 56.2 % of participants, while 25.2 % exhibited levels of insufficiency. A higher BMI was associated with lower vitamin D levels, whereas daily supplementation correlated with higher levels compared to intermittent or weekly dosing regimens. The vast majority of patients expressed high satisfaction with the services provided. Pharmacists valued the positive feedback from patients and expressed enthusiasm for further testing and the advancement of clinical pharmacy services. The resulting maturity matrix facilitates corporate implementation.

**Conclusion:**

The implementation of a large-scale federal vitamin D screening campaign proved to be feasible and resulted in high levels of satisfaction among both patients and providers. The findings indicated significantly low vitamin D levels among participants. Pharmacists expressed a desire for an expansion of clinical pharmacy services in the future.

Vitamin D testing in pharmacies: results of a federal screening campaign.

## 1 Introduction

With the shift towards patient centered services, pharmacies around the world are expanding their careaccording to local needs.^1^, ^2^, ^3^, ^4^ Community pharmacies represent an optimal setting for screening and prevention initiatives due to their accessibility and the substantial patient population they serve.^5^ Globally, pharmacies frequently provide screening services such as blood pressure monitoring and blood glucose testing; however, these services are seldom integrated into comprehensive healthcare strategies, with public support and interprofessional collaboration.^6^ Additionally, during the SARS-CoV-2 pandemic, pharmacies implemented rapid and PCR point-of-care testing (POCT) for COVID-19 as part of their diagnostic screening offerings.^7^ In a study of Hohmeier et al. on influenza testing in community pharmacies, it was shown that successful implementation of POCT requires public awareness, pharmacist acceptance, leadership support, and support of health providers external to the pharmacy.^8^ Abdellatife et al. found reimbursement, patient needs and relevanceas important factors for implementation of POCT in acute respiratory infections.^9^ Despite harsh criticism by medical associations, Austrian legislation expanded pharmacy services to include medication reviews and diagnostic testing by nasopharyngeal swab tests and capillary blood samples in February 2024, enabling POCT and screening campaigns. Pharmacies, wishing to conduct POCT need to register this service at their regional health department.

In recent years, there has been a growing public awareness regarding vitamin D, as various health conditions and parameters have been associated with vitamin D deficiency, albeit often with limited empirical support.^10^ Conversely, low levels of vitamin D have been linked to compromised bone health, mood disorders and an elevated risk of falls and frailty.^11^, ^12^, ^13^ Pharmacists have demonstrated their capability in vitamin D POCT in recent studies. Busutill et al. tested the vitamin D levels of 80 participants using a POCT in pharmacies.^14^ The POCT method demonstrated a high reliability and more than 50 % of participants had a vitamin D deficiency in this study. Accuracy of vitamin D POCT analyzers was also established by Albrecht et al. and Yett et al^15^^,^^16^ In light of the recent legislative changes that enhance the role of pharmacists in healthcare, the pharmacy board of Salzburg, Austria, has launched a vitamin D POCT screening campaign as a pilot project, commencing in April 2024. The board initiated the campaign and provided the immunoassay instrument upon enrollment. Three local meetings were offered with detailed information on the campaign for interested pharmacists. This included background knowledge on pharmacotherapy, as well as information about the campaign, the implementation of POCT, the study's purpose, and how to participate.

## 2 Aims

The aim of this study was to quantify the results of the vitamin D screening campaign regarding the vitamin D levels of the population, as well as to explore patient and provider satisfaction with the implementation of the novel clinical pharmacy service. A maturity matrix for implementation of vitamin D testing in community pharmacy was also developed based on the results.

## 3 Methods

### 3.1 Study design

The study was a cross-sectional multicenter survey. Community pharmacies were invited twice in January 2024 by the board of pharmacy of Salzburg via the regular newsletter to participate in the screening project.

Ethics.

A full ethics waiver was granted by the regional ethics committee of the state of Salzburg due to the anonymous nature of the questionnaires. The intervention was regarded as part of standard care in Austrian Pharmacies, the data was collected and provided anonymously by the board of pharmacy to the researchers. The study was registered at the German Clinical Trials Register (DRKS00034147).

Vitamin D testing.

### 3.2 Recruitment of pharmacies

**Fig. 1:**
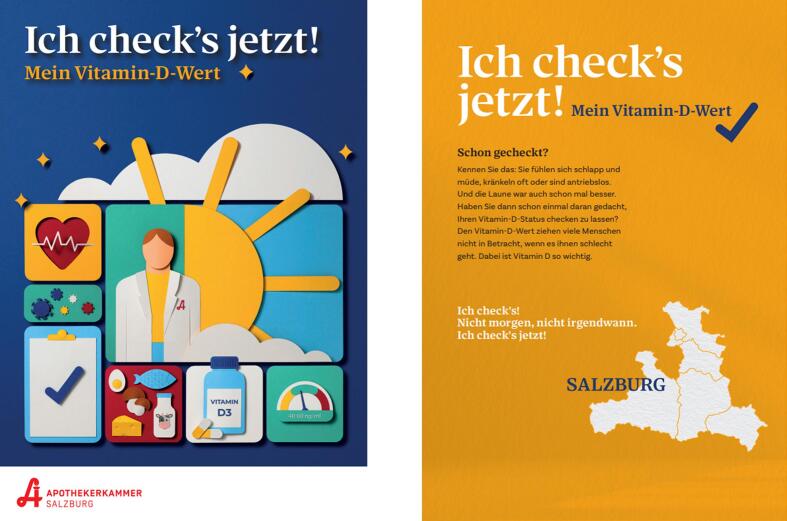
Images of the campaign, placed in pharmacies, newspapers and on social media.

Pharmacies were recruited through printed materials, emails, and local meetings, all organized by the board of pharmacy. The population of Salzburg state was informed of the campaign by newspaper, social media posts and posters inside of the pharmacies (Fig. 1). The screening campaign was endorsed by the federal ministry of health. The Salzburg state minister of health attended the joint kick-off press conference on April 8, 2024. Testing in pharmacies was offered between April 8 and April 20, 2024 and performed by registered pharmacists.

### 3.3 Materials

The measurement in all pharmacies was conducted with the “Automatic Fluorescent-Immunoassay-System” (AFIAS-1) of Boditech Med Inc., which is a single channel lateral flow immunoassay platform. The integrated automatic pipetting system allows easy handling and no further sample preparation is required. An amount of Thirty μL of blood from the fingertip is used and, the test result is available in approximately 12 min. Vitamin D levels above 100 ng/ml and lower than 4 ng/ml were not able to be calculated accurately and were instead displayed as >100 mg/ml and < 4 ng/ml, respectively. The AFIAS-1 device was offered to participating pharmacies by the board of pharmacy for free during the screening period. Expendable materials, including the test kit, was purchased by the pharmacy. Patients paid 9.50 € for each test.

### 3.4 Patient questionnaire

The patient questionnaire was developed based on the framework of Boynton et al., which provided guidance on questionnaire design and validity, among other aspects.^17^ Domains were created to collect the following content:

1. Baseline data, including age group, gender, height and weight, health status, time spent outdoors and extreme sun exposure over the previous two to three months.
2. Items pertaining to vitamin D. These items addressed the perception of vitamin D for personal health, the attitude towards of vitamin D screening in general and vitamin D intake.
3. Items regarding the screening project. Awareness of the project, personal motivation for testing, the patient's knowledge about testing in pharmacies and whether the patient had previously undergone a vitamin D test.
4. Items pertaining to customer satisfaction with the testing service on a 5-point Likert scale. These items addressed perceived hygiene and safety, the technique of measurement, appointment scheduling and waiting time, the competence of the performing person, and information and counselling in relation to testing and vitamin D supplements.
5. An open-ended question at the end of the questionnaire to provide further comments on the project.

Content validation was established through a pilot with five pharmacists who were trained on point of care testing (POCT) and vitamin D supplementation before. This led to minor changes in the questionnaire including, height and weight of the patient were added, the question on sun exposure was rephrased, the question about time spent outside was split into working and workfree days, the questions on perceived importance of vitamin D and vitamin D tests were rephrased, and a question on the patient's most recent vitamin D intake was added. Face validation of the questionnaire established through a pilot with five potential participants. This validation step did not lead to changes in the questionnaire. The final patient questionnaire was sent to the participating pharmacies by the board of pharmacy in printed format and was also made available for download. The single paper questionnaires were handed out to participants after vitamin D testing, together with the result of the test. Patients completed the questionnaire anonymously and dropped it to a provided poll box.

By end of April, the patient questionnaires were sent by the pharmacies to the board of pharmacy in a pre-paid envelope. To facilitate digitization, an identical online questionnaire was created, using Microsoft® forms. All paper questionnaires were pseudonymized and manually entered into the online questionnaire. For analysis, Microsoft® forms exported the data into an Excel® spreadsheet, including all completed questionnaires. For all fields that were not filled by the patients, the word ‘not available’ was entered.

### 3.5 Pharmacist questionnaire

The questionnaire for pharmacists was developed in a similar fashion as the patient questionnaire. Initially four different domains with regard to vitamin D testing (testing procedure and implementation barriers, initial instructions, perceived feedback and free text feedback) were created. Additionally, four different domains with regard to new clinical pharmacy services were created (POCT, medication review, vaccinations, general questions). The link to the electronic questionnaire for pharmacists was sent to participating pharmacies via email one week after the campaign ended, directly from the board of pharmacy. A follow-up reminder was sent after another week. The questionnaire was developed using Microsoft® forms.

### 3.6 Statistical analyses

As there is no universal grading system, vitamin D clusters were formed according to Wacker et al., Spiro et al., Hansen et al. and Holick et al.^18^, ^19^, ^20^, ^21^ with 0–11.99 ng/ml indicating severe vitamin D deficiency, 12.00–19.99 ng/dl as deficiency, 20.00–29.99 ng/ml as insufficiency, 30.00–39.99 ng/ml as sufficient, 40.00–59.99 ng/ml as optimal range, 60.00–89.99 ng/ml as high and > 90.00 ng/ml as too high. Subclasses of patients were formed and analyzed regarding gender, age, health state, time spent outside, frequency of vitamin D intake and sun exposure during the past 2–3 months.

Quantitative data were analyzed descriptively, using Microsoft® Excel® 2021. Jamovi 2.3.28 was used for statistical testing and creating scatter- and violin plots. The Mann-Whitney *U* test was used for significance testing of nonparametric data, specifically to assess differences between male and female participants. The Pearson correlation was used for parametric testing of the body mass index (BMI) in relation to vitamin D levels. Missing data were reported but patients were still included (analysis with missing data). Patient groups were formed (SE) and double checked by two other researchers (SC, OR) based on the data and summary key descriptive measures for each age group were calculated, such as mean ± standard deviation (SD). Qualitative data were systematically categorized into three main themes: positive aspects, barriers, and precursors. To ensure a structured analysis, all responses were compiled into a dedicated Microsoft® Excel® 2021 file. Each response was carefully reviewed (SE, SC, OR) and assigned to one of the three categories based on its content. A color-coding system was applied to visually differentiate the themes, enhancing clarity and organization. This approach facilitated efficient data handling and allowed for the identification of recurring patterns and key insights within the responses.

### 3.7 Maturity matrix

The concept of a maturity matrix for community pharmacies has been adopted by Teichert et al. in 2024 from original approaches in orthopedic surgery and primary care. The intention was to develop a tool to self-assess organizational readiness and support implementation of clinical pharmacy services.^22^, ^23^, ^24^ The Teichert matrix consists of five different facilitator domains and four growth steps for each domain. The growth step “performing, evaluating and improving” has been divided into the two steps of “performing” and “evaluating and improving” in this study, as data on these steps was generated with separate questions. The fields of the matrix were filled with the study findings from the pharmacist questionnaire and with general insights gained during the campaign. In all cases, the items were developed in an iterative way through three meetings of the study authors, where consensus was made. The first meeting was on organizational aspects, including delivery of devices, information on standard operating procedures and creating awareness on the campaign. Members of the board were included to learn from their experience. The second meeting was on performance, the roll out and how to collect data for the study. In a third meeting the campaign was evaluated, including feedback from the pharmacists, following the consolidated framework of Damschroder et al^25^ The matrix aims to be used by pharmacies as a guidance for implementation.

## 4 Results

### 4.1 Vitamin D screening

The screening program was offered state-wide by 62 out of 94 community pharmacies (66 %), covering all districts. During the two-week span, 2770 patients enrolled and were screened for their vitamin D level (a mean of 45 tests per pharmacy). Females and older patients were clearly overrepresented. Table 1 shows the patient demographics in detail.

**Table 1:** Demographics of screened patients.

Parameter		N=	% of total
Gender	Female	2090	75.5
	Male	674	24.3
Age (y)	18–24	148	5.3
	25–34	212	7.7
	35–49	400	14.4
	50–64	948	34.2
	65+	1041	37.6
	missing data	21	0.8

All retrieved 2770 questionnaires contained vitamin D levels and could be used for analyses. There were no dropouts. Vitamin D levels were significantly higher for female than for male participants (*p* < 0.001). Vitamin D deficiency, defined as levels below 30 ng/dl were measured in 56.2 % of participants, and levels <20 ng/ml in 25.2 % of participants. Table 2 shows the vitamin D levels of the whole sample and of the subgroups gender, age, health state, time spent in sunlight, frequency of dosing and forced sun exposure.

**Table 2:** Results of vitamin D testing by subgroups.

Cluster	Parameter	N=	Mean vit. D (ng/ml)	SD
Gender (*n* = 2639)	female	2090	32.9	17.9
	male	674	29.3	17.2
	without	6	26.7	15.0
	total	2770	32.0	17.8
Vit. D level cluster (ng/ml, *n* = 2770)	4.00–11.99	154		
	12.00–19.99	544		
	20.00–29.99	859		
	30.00–39.99	535		
	40.00–59.99	466		
	60.00–89.99	160		
	>90	52		
Age (years) (n = 2770)	18–24	148	23.4	13.0
	25–34	212	27.8	17.0
	35–49	400	29.4	14.8
	50–64	948	33.1	17.4
	65+	1041	34.1	19.4
	missing	21	34.6	15.0
Health state (*n* = 2708)	healthy	2256	31.8	17.5
	chronic diseases	417	33.3	18.8
	acute disease	35	40.8	23.3
	missing	62	27.6	14.4
Time spent outside (working days) (*n* = 2770)	0–10	144	29.7	16.3
	20	382	30.3	17.2
	30–50	900	31.8	17.4
	60–90	639	33.6	19.6
	> 2 h	475	31.7	16.8
	missing	230	33.7	17.6
Time spend outside (days off work) (n = 2770)	0–10	27	29.5	19.8
	20	89	27.8	17.3
	30–50	466	31.3	18.3
	60–90	843	31.9	17.8
	> 2 h	1277	32.5	17.5
	missing	68	35.6	20.3
Frequency of vitamin D intake (*n* = 2838)	daily	565	41.3	22.1
	several times a week	302	38.3	18.8
	once a week	590	35.5	16.7
	rarely	614	27.4	13.4
	never	655	22.2	11.2
	missing	44	34.6	21.7
Particular sun exposure during past 2–3 months (n = 2770)	none	1634	31.1	17.5
	beach holiday/holiday in the sun for at least 2 weeks;	113	30.9	18.4
	frequent sunbathing or solarium;	71	34.1	17.3
	frequent skiing or equivalent	677	33.3	17.5
	frequent skiing or equivalent for at least 2 weeks;	51	36.4	19.9
	frequent skiing and frequent sunbathing or solarium	20	33.4	17.2
	others	179	34.2	20.6
	missing	25	33.2	18.3

A scatter plot was generated to investigate the correlation between BMI and vitamin D level. Vitamin D levels were clustered into seven groups, according to deficiency grades (x-axis). The mean BMI (y-axis) for each vitamin D group was calculated and plotted (Fig. 1). The generated diagram shows a descending trendline, which displays the significantly negative correlation between vitamin D levels and BMI (Pearson's R − 0,984; *p* < 0.001), indicating that individuals with a higher BMI tend to have lover levels of vitamin D.

Vitamin D levels clustered as: 0–11.99 ng/ml indicating severe vitamin D deficiency (group 1), 12.00–19.99 ng/dl as deficiency (group 2), 20.00–29.99 ng/ml as insufficiency (group 3), 30.00–39.99 ng/ml as sufficient (group 4), 40.00–59.99 ng/ml as optimal range (group 5), 60.00–89.99 ng/ml as high (group 6) and > 90.00 ng/ml as too high (group 7).

Patient questionnaire.

**Fig. 2:**
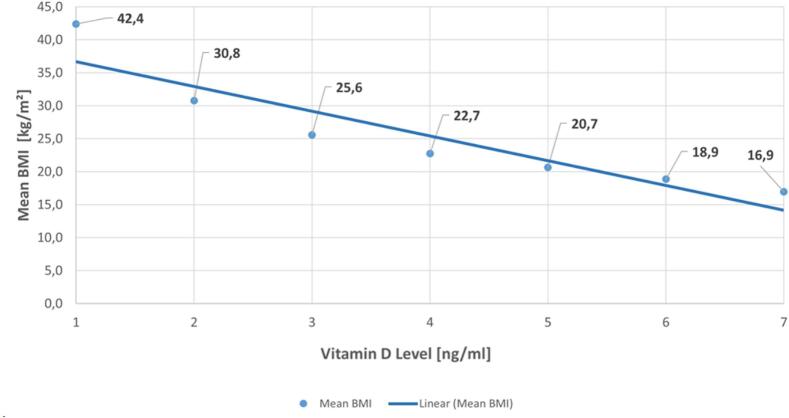
Scatter plot: correlation of BMI and vitamin D levels.

Mean levels of vitamin D were calculated based on the dosing intervals reported by patients (daily, several times per week, once a week, rarely and never). Fig. 2 shows that the less frequently the dosing was scheduled, the lower the vitamin D levels (Pearson's R − 0.980; *p* = 0.003). The (cumulative) vitamin D dose was not included, as patients could not remember the dose during pretesting of the questionnaire. The violin plot shows the distribution of the data.

When asked about the importance of vitamin D testing, 2114 of 2717 patients (76.3 %) strongly agreed with the statement “in my opinion, vitamin D is very important for health”. Numbers were higher for females with 79.9 % of strong agreement to the importance of vitamin D in contrast to males (68.3 % of strong agreement). Similarly high numbers were found for the perceived importance of vitamin D testing (70.7 % of participants strongly agreed, 73.6 % of women versus 61.9 % of men, *p* > 0.05).

The manner in which participants learned about the project was assessed by 2716 individuals. The majority of patients reported that they were informed about the campaign through their pharmacy, while a smaller proportion cited family and friends, printed media, social media, or television as their primary sources. A detailed overview of the distribution of information sources is presented in Table 3.

**Table 3:** Results on how patients were informed about the project (*n* = 2716).

How did you find out about the project?	N=	% of Total	Cumulative %
In the pharmacy	1052	38.0 %	38.0 %
Family & friends	545	19.7 %	57.7 %
Printed media	492	17.8 %	75.5 %
Social media/TV	416	15.0 %	90.5 %
Printed media and social media/TV	31	1.1 %	91.6 %
Printed media and in the pharmacy	21	0.8 %	92.4 %
Printed media and family & friends	21	0.8 %	93.2 %
Social media/TV and in the pharmacy	23	0.8 %	94.0 %
In the pharmacy and family & friends	14	0.5 %	94.5 %
Social media/TV and family & friends	11	0.4 %	94.9 %
Printed media and in the pharmacy and family & friends	2	0.1 %	95.0 %
Printed media and social media/TV and family & friends	3	0.1 %	95.1 %
Social media/TV and in the pharmacy and printed media and family & friends	3	0.1 %	95.2 %
Others	81	2.9 %	98.1 %
Missing	54	1.9 %	100.0 %

Nearly half of patients (*n* = 1295, 46.8 %) stated they had never had their vitamin D level tested before. One-fifth of participants reported having had a vitamin D deficiency previously (*n* = 565, 20.4 %). Four out of five patients (80.8 %, *n* = 2237/2722) stated that they didn't expect that such a test was performed in a pharmacy. Those who had their vitamin D level tested before had higher mean levels (36.0 ng/ml) than those, who didn't (29.6 ng/ml).

**Fig. 3:**
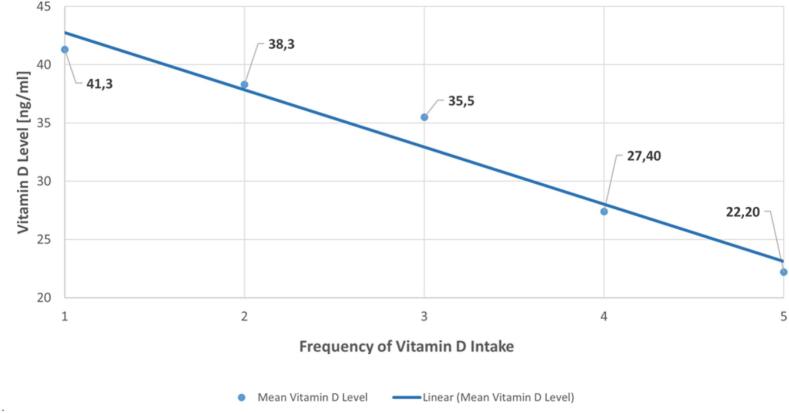
Scatter plot of dosing interval and vitamin D levels. Frequency of Vitamin D intake clustered as: 1 = daily, 2 = several times a week, 3 = once a week, 4 = rarely, 5 = never.

Almost all patients strongly agreed that high standards of hygiene and safety were maintained during vitamin D testing in the pharmacy (*n* = 2580/2676, 93.1 %). Numbers for satisfaction with measuring technique, timing and waiting time were similarly high (93.0 % strongly agreed), ratings for patient information and the individual pharmacists' competencies ranked even higher (94.0 % resp. 96.0 % strongly agreed). Patients' written comments were mainly positive (Fig. 3).

### 4.2 Pharmacist questionnaire

Most pharmacists who participated responded to the follow-up questionnaire on the screening campaign and gave their appraisal on future clinical pharmacy services (45 out of 62 pharmacists, 84.4 % female). This comes close to the 86.7 % of female pharmacists in greater Austria.^26^ They were quite experienced (female 16.2. years of experience, SD 11.14, male 20 years, SD 6.11) and the mean age was 42.3 years (SD 11.3) for female pharmacists and 47.9 years (SD 5.93) for male pharmacists. Most pharmacists stated that the campaign went smoothly, that they had the time, personnel and spatial resources and that patient feedback was mainly positive. Information on the campaign and instructions on the device were rated more critically. Most pharmacists would like to continue testing (87 %) and perform vaccinations (78 %) and medication reviews (91 %) in the future. Pharmacists, who stated that they would like to continue POCT were asked, which parameter they would prefer to test next. Feedback was given from 39 pharmacists (86.7 % of all participating pharmacists). They mentioned blood sugar and micronutrients (*n* = 35, 77.8 %), lipids (*n* = 33, 73.3 %), communicable diseases (influenza A/B, RSV, COVID 19, etc., *n* = 24, 53.3 %), vaccination titer testing (*n* = 21, 46.7 %), C-reactive protein (CRP, *n* = 20, 44.4 %), inflammation parameters (*n* = 18, 40.0 %) and one pharmacist opted for prostate specific antigen (PSA, 2.22 %).

The overwhelming majority supports a professional change towards more clinical pharmacy services (60 % agree strongly, 24 % rather agree). It is noteworthy to mention that the majority of pharmacists feels only partly prepared to start medication reviews (53 %) or to initiate a treatment plan, based on testing results (55 %). Detailed data on the pharmacists' questionnaire can be found in Table 4.

**Table 4:** Results of the questionnaire for participating pharmacists.

Questions to pharmacists	Parameter	N=	% of total
Overall, performing the test went well.	Strongly agree	22	48.9 %
	Somewhat agree	12	26.7 %
	Neither agree or disagree	7	15.6 %
	Somewhat disagree	3	6.7 %
	Strongly disagree	1	2.2 %
My pharmacy had the time and personnel resources available for testing.	Strongly agree	18	40.0 %
	Somewhat agree	19	42.2 %
	Neither agree or disagree	6	13.3 %
	Somewhat disagree	1	2.2 %
	Strongly disagree	1	2.2 %
The spatial resources for testing in my pharmacy were available.	Strongly agree	30	66.7 %
	Somewhat agree	8	17.8 %
	Neither agree or disagree	3	6.7 %
	Somewhat disagree	3	6.7 %
	Strongly disagree	1	2.2 %
There was positive feedback from my patients.	Strongly agree	35	77.8 %
	Somewhat agree	10	22.2 %
There was negative feedback from my patients.	Neither agree or disagree	2	4.4 %
	Somewhat disagree	9	20.0 %
Patients responded well to my advice on the test results.	Strongly agree	34	75.6 %
	Somewhat agree	10	22.2 %
	Neither agree or disagree	1	2.2 %
Would you like to offer this and other tests in your pharmacy in the future?	Yes	39	86.7 %
	No	4	8.9 %
	Missing	2	4.4 %
Would you like to offer and carry out medication reviews in your pharmacy in the future and complete the relevant training courses?	Yes	41	91.1 %
	No	1	2.2 %
	Missing	3	6.7 %
Do you feel well trained and prepared to perform medication reviews?	Yes	14	31.1 %
	Neither agree or disagree	24	53.3 %
	No	6	13.3 %
	Missing	1	2.2 %
Pharmacists should select the right patients for a medication review and should be reimbursed for this service.	Strongly agree	17	37.8 %
	Somewhat agree	19	42.2 %
	Neither agree or disagree	4	8.9 %
	Somewhat disagree	1	2.2 %
	Strongly disagree	3	6.7 %
	Missing	1	2.2 %
I would welcome using a supporting software for medication management to save time.	Strongly agree	37	82.2 %
	Somewhat agree	5	11.1 %
	Neither agree or disagree	2	4.4 %
	Missing	1	2.2 %
Vaccinations in pharmacies can increase the vaccination rate in the future.	Strongly agree	31	68.9 %
	Somewhat agree	5	11.1 %
	Neither agree or disagree	5	11.1 %
	Somewhat disagree	2	4.4 %
	Strongly disagree	2	4.4 %
Have you already completed the relevant vaccination training at the board of pharmacy?	Yes	28	68.3 %
	No	13	31.7 %
Would you offer a vaccination service in your pharmacy in the future if it were adequately remunerated?	Yes	35	77.8 %
	No	7	15.6 %
	Missing	3	6.7 %
Due to their accessibility, pharmacies will be a suitable place for vaccinations in the future.	Strongly agree	28	62.2 %
	Somewhat agree	9	20.0 %
	Neither agree or disagree	2	4.4 %
	Somewhat disagree	1	2.2 %
	Strongly disagree	2	4.4 %
	Missing	3	6.7 %
Due to their accessibility, pharmacies will be a suitable place for clinical pharmacy services in the future.	Strongly agree	28	62.2 %
	Somewhat agree	13	28.9 %
	Neither agree or disagree	2	4.4 %
	Somewhat disagree	1	2.2 %
	Missing	1	2.2 %
My pharmacy has spatial resources to offer clinical pharmacy services	Strongly agree	19	42.2 %
	Somewhat agree	8	17.8 %
	Neither agree or disagree	10	22.2 %
	Somewhat disagree	4	8.9 %
	Strongly disagree	3	6.7 %
	Missing	1	2.2 %
In our pharmacy, we are very concerned about implementation of clinical pharmacy services, as we expect complaints by physicians.	Strongly agree	3	6.7 %
	Somewhat agree	8	17.8 %
	Neither agree or disagree	19	42.2 %
	Somewhat disagree	11	24.4 %
	Strongly disagree	2	4.4 %
	Missing	2	4.4 %
Pharmacies should have full access to the electronic patient record.	Strongly agree	27	60.0 %
	Somewhat agree	8	17.8 %
	Neither agree or disagree	8	17.8 %
	Somewhat disagree	1	2.2 %
	Missing	1	2.2 %
I favor a change in pharmacies towards more clinical pharmacy services.	Strongly agree	27	60.0 %
	Somewhat agree	11	24.4 %
	Neither agree or disagree	4	8.9 %
	Somewhat disagree	1	2.2 %
	Strongly disagree	1	2.2 %
	Missing	1	2.2 %
I feel prepared to initiate treatment based on the results of testing (such as UTI) and medication reviews.	Strongly agree	12	26.7 %
	Somewhat agree	15	33.3 %
	Neither agree or disagree	10	22.2 %
	Somewhat disagree	2	4.4 %
	Strongly disagree	5	11.1 %
	Missing	1	2.2 %

**Fig. 4:**
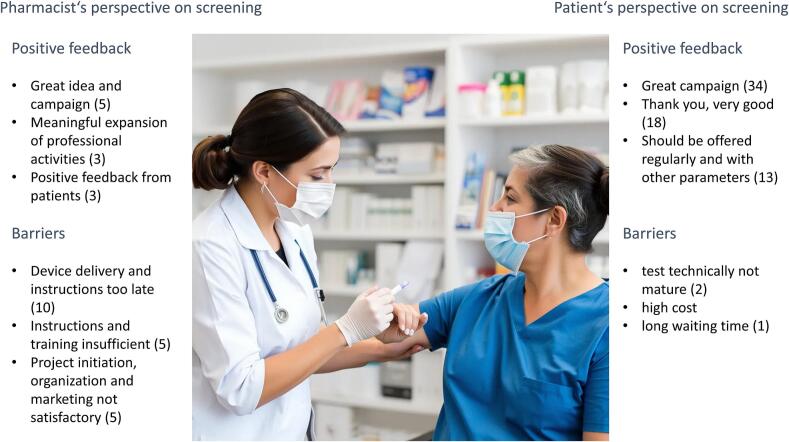
Clustered qualitative feedback on the campaign.

Open text feedback from pharmacists was mainly positive, with some complaints on the late information and delivery of the devices (Fig. 4).

Additional feedback from the pharmacists was provided on clinical pharmacy services in the free text field:

1. “Pharmacies must have time to grow into this new role.” (female, 57 years, 30 years of working experience)
2. “The conflicts between the chambers of pharmacists and the chamber of physicians regarding this topic mostly take place at levels that have little to do with actual on-site practice. The relationship with our local physicians is almost entirely very respectful and on equal terms, I would never want to endanger that, we just need to pool our expertise and talk to each other (already a lived practice in a Salzburg project in nursing homes involving doctors, pharmacists and nursing staff).” (female, 58 years, 33 years of working experience)
3. “It is welcomed if we can offer more services in pharmacies, albeit remunerated by the health insurance funds at the same rate as physicians.” (female, 58 years, 34 years of working experience)
4. “I believe that the future of the pharmacy is to offer clinical-pharmacy services” (female, 60 years, 35 years of working experience)
5. “Vaccination has no part to play in pharmacies. This is an intervention in the body and should remain in the hands of physicians!” (female, 40 years, 15 years of working experience)

### 4.3 Maturity matrix

The maturity matrix, created from the study results, provided the framework for service implementation and evaluation of pharmacists' readiness for vitamin D screening. The matrix cells were filled with the study findings (Table 5).

**Table 5:** Maturity matrix for the implementation of a vitamin D screening.

Maturity Matrix for vitamin D screenings in pharmacies	Personalized care	Teamwork	Information systems and data exchange	External collaboration	Education and research
Being aware and motivated	• Identify patient groups, for which screening makes sense • Create awareness for prevention • Provide education on thresholds and pharmacotherapy	• Vitamin D training at team meetings • Sensitizing team members to high-risk patients and risks associated with vitamin D use • review individual performance with the team, pay attention to the skills required for vitamin D screening and counselling	• Raise awareness on the importance of vitamin D screening • Know the measurement technique • Know which patients need further testing or evaluation by physicians • Raising awareness for the registration of relevant patient characteristics	• Identify related healthcare providers in vitamin D care (medical, nutritional, therapeutical)	• Investigate in new test methods • Offer training to students, pharmacists, and other health care providers • Know current guidelines and trends in vitamin D supplementation
Being able to	• Counselling competence • Providing information material for patients • Offer individual counselling for patients	• Plan and discuss the screening activity within the team	• Communicate the campaign to other healthcare providers • Consent on consistent counselling and recommendations on how to cope with vitamin D deficiency	• Allocate tasks to each team member • Arrange ways to make appointments (phone, web-based, etc.) • Support adherence	• Search for opportunities to provide training • Investigate in pharmacotherapy and pharmacy practice
Performing	• Support patient self-management • Provide personalized advice on vitamin D supplementation and sun protection • Document test results	• Implementation of team training on vitamin D supplementation • Carrying out consultations for the initial and follow-up treatment of vitamin D preparations	• Work according to a protocol if previous measurement results are not available • Referrals to the physician • Make equipment and test materials available	• Advices on alternative sources of vitamin D • Share experience with other pharmacists • Offer collaborative consultations with GP and pharmacist	• Conduct training on vitamin D and vitamin D deficiency
Evaluating and improving	• Evaluation of patient satisfaction • Documentation of screened numbers and levels • Offering retesting, for example after supplementation	• Discussion of evaluation during team meetings • Addressing issues and improvements • Evaluation of teamwork	• Enter test results to (electronic) documentation system	• Evaluate (and improve) interprofessional collaboration	• Collect feedback and evaluate training programs
Innovating	• Offer regular vitamin D testing (2×/year) • Reflect own performance with colleagues	• Offer screening campaigns • Select a responsible team member for testing	• Find support systems • Increase adherence by automated reminders	• Cooperation with other healthcare providers • Collaborate with general practitioners	• Expand service to further tests • Attend medical education courses

## 5 Discussion

A federal vitamin D screening project in pharmacies was conducted and evaluated just after a new law permitted clinical pharmacy services. In addition, patient satisfaction and and pharmacists' perception of vitamin D testing and other clinical services were explored. Previous population-based studies on vitamin D did not describe any community pharmacy based screening campaigns.^27^

Using the 2012 Austrian Nutrition Report, Spiro et al. found vitamin D deficiency (defined as <25 ng/ml) in 11.6 % of adult female Austrians between 18 and 64 years of age, and 14.2 % of their male counterparts.^19^ Vitamin D deficiency was more frequent in older individuals (>65 years of age) across both females (19.9 %) and males (20.4 %). This current screening campaign found higher levels of vitamin D deficiency, differently defined as levels below 30 ng/dl in 56.2 % of participants, and 25.2 % of participants with levels <20 ng/dl. The higher rate of vitamin D deficient patients could be due to a selection bias, where greater numbers of patients with a higher probability of vitamin D deficiency chose to participate compared to the prior study, or due to the campaign timing in April, when vitamin D levels are usually lower than other times of the year.^28^ In a similar screening study on healthy adult Austrians, Kudlacek et al. found significant vitamin D deficiency 25 years ago, which is in line with our results.^29^

Even though there is little evidence of benefits from high supplementary vitamin D doses,^30^ there is consensus that levels <30 ng/ml should be corrected.^31^ This would apply to more than half of the tested individuals in this study. It can be assumed that vitamin D levels have been similarly low in the preceding months at wintertime, as modelled by O'Neill et al. for different European latitudes and proposing a vitamin D winter for Austria from November to March.^32^ However, routine vitamin D screening is controversial. The 2024 US Endocrine Society guideline drew much attention by suggesting against vitamin D screening.^33^ This statement caused a clinical discussion, as there are several subgroups of patients, where low vitamin D levels can be expected and might be harmful, like inflammatory diseases, people working nightshifts, people who cover their skin for religious purpose or to protect the skin for other reasons. Extra-skeletal effects, like the clear correlation between vitamin D levels and periodontitis, or its effect on the immune system need to be taken into account.^31^^,^^34^ These are all good reasons to endorse vitamin D screenings despite the recommendation of the US Endocrine Society and the non-consensus. The high number of deficient and insufficient vitamin D levels found in this screening campaign can further support screenings. Screening in pharmacies, however, might already represent a selection bias towards subgroups with lower vitamin D levels and may therefore differ positively from screening in the general population. The clear correlation between high BMI and low vitamin D levels was another interesting finding. Although it was previously shown that vitamin D levels are lower in obese individuals, the large number of tests allowed a clear BMI correlation for non-obese to be revealed.^35^ The positive effects of daily dosing versus other dosing regimens on vitamin D levels were unexpected and not described before. The methodology relies on self-reporting, based on the assumption that patients may not accurately remember the precise dosage but can recall the dosing interval. A meta-analysis by Zhuang et al. in contrast found no difference between daily and weekly intervals, recommending the more convenient weekly dosing.^36^ As adherence tends to be better with weekly than with daily dosing and large doses are usually effective, the implications of this result remains unclear.^21^^,^^37^ As this new data supports daily vitamin D dosing, it needs to be considered that the study was not designed to distinguish between the dosing frequencies and dosages were not included. That means that patients might even have taken the daily dose only once a week.

Patient satisfaction with testing in the pharmacies was very high in this study. This can be attributed to the easy accessibility of pharmacies and high trust in pharmacists. Results are in line with former studies on clinical pharmacy services like vaccinations,^38^ and are confirmed by a review by Yuliandani et al., stating similar high levels of patient satisfaction throughout multiple clinical pharmacy services.^39^ As most patients were informed about the campaign in pharmacies, and considering the lower cost-effectiveness of advertising these services outside of pharmacies, this channel may be preferred for future screening campaigns.

Pharmacists' feedback about the project was mainly positive, with the exception of a partly overhasty coordination and in some cases delayed delivery and instruction on the fluorescent-immunoassay-test-system. Participating pharmacists stated their interest in further POCT and clinical pharmacy services. This is in line with data from almost all countries during this time of professional transformation.^38^^,^^40^^,^^41^ However, many pharmacists stated they feel inadequately prepared to perform medication reviews or initiate a medication regimen based on test results, deficits in pharmacotherapy are present here. Pharmacists clearly favored future professional development towards more clinical pharmacy services but again didn't feel adequately prepared for the shift to a similar degree. This statement can be understood as a signal for the national board of pharmacy and for the Ministry of Health to support further changes, and provide more training in pharmacotherapy along with implementation.

In Austria, this vitamin D screening initiative represents one of the first widespread cognitive pharmacy services, launched by the pharmacy board, with backing from the Ministry of Health and assessment by academic researchers. The pharmacy board regarded the substantial participant turnout as a motivating factor for the continuation of screening initiatives. The Ministry of Health has prioritized preventive measures in its agenda and, drawing from preliminary findings, has indicated a strong interest in future partnerships with pharmacies to enhance public health outcomes. Insights gathered from the participating pharmacists can inform the refinement of subsequent campaigns, while their favorable perceptions may facilitate the extension of similar initiatives into additional clinical pharmacy services. The provided maturity matrix can be utilized by individual pharmacies to check preparedness of the own facility to conduct POCT and can easily be transferred to other clinical pharmacy services.

### 5.1 Strengths and limitations

The screening campaign successfully engaged a substantial number of participants. Vitamin D levels were assessed from a diverse sample encompassing urban, suburban, and rural regions, which span altitudes ranging from 378 to 3657 m above mean sea level, thereby reflecting varying intensities of UV radiation. The participating pharmacies accounted for two-thirds of the total pharmacies in Salzburg.

Although participants completed the survey anonymously and submitted it into a collection box, the presence of pharmacy staff may have influenced their responses. Pharmacists' perspectives on the future of professional development may be subject to selection bias, as these pharmacists were likely those with a vested interest in clinical pharmacy services. Additionally, a selection bias may also be present within the screened population. Participants reported that they primarily learned about the campaign through pharmacies, suggesting that patients with pre-existing health conditions may be disproportionately represented in the sample. The study cohort exhibits a predominance of female participants, comprising 75.5 % of the sample, in contrast to the national average in Austria, which stands at 50.6 %.^42^ Additionally, 71.8 % of the participants are aged 50 years or older, whereas the overall percentage for this age group in Austria is approximately 33 %. Consequently, the baseline characteristics of the study population are skewed towards a higher representation of female and elderly individuals.

## 6 Conclusions

A comprehensive vitamin D screening initiative conducted in pharmacies demonstrated feasibility, appeal, and significant participant engagement. Pharmacies, due to their extensive daily interactions with customers, effectively reached patients when supplemented with promotional materials and public endorsements. The findings revealed unexpectedly low vitamin D levels, with over half of the participants exhibiting levels below the 30 ng/ml threshold, thereby necessitating recommendations for supplementation. Notably, vitamin D deficiencies were more prevalent among individuals with higher BMIs. The study indicated that daily dosing of vitamin D proved to be more efficacious than alternative dosing regimens. Participants expressed satisfaction with the convenience of testing in a pharmacy setting, appreciating the accessibility and high medical standards maintained. Pharmacists reported a positive experience with the campaign and received favorable feedback from patients regarding their services. Pharmacists expressed support for continued testing and professional development aimed at enhancing clinical pharmacy practices, while also indicating a need for improved preparedness and education in pharmacotherapy. The resulting maturity matrix offers a framework for community pharmacies to implement vitamin D screenings and may be applicable to other POCT initiatives. However, the generalizability of these findings to other geographical regions with varying latitudes and jurisdictions remains uncertain, particularly concerning the implementation of clinical pharmacy services and the education of pharmacotherapy.

## CRediT authorship contribution statement

**Olaf Rose:** Writing – review & editing, Writing – original draft, Visualization, Validation, Supervision, Software, Resources, Project administration, Methodology, Investigation, Formal analysis, Data curation, Conceptualization. **Stefanie Eppacher:** Writing – review & editing, Writing – original draft, Visualization, Validation, Software, Investigation, Formal analysis, Data curation, Conceptualization. **Johanna Pachmayr:** Writing – review & editing, Resources. **Stephanie Clemens:** Writing – review & editing, Visualization, Validation, Supervision, Software, Resources, Project administration, Methodology, Investigation, Formal analysis, Data curation, Conceptualization.

## Funding

This research did not receive any specific grant from funding agencies in the public, commercial, or not-for-profit sectors.

## Declaration of competing interest

No potential competing interest was reported by the authors.

## Appendix A Supplementary data

Categorized feedback from patients. Image 1

Categorized feedback from pharmacists. Image 2
