# Supplementary material for: Vitamin D testing in pharmacies: Results of a federal screening campaign
Source: Explor Res Clin Soc Pharm. 2025 Mar 25;18:100596. doi: 10.1016/j.rcsop.2025.100596 (PMC11997348; doi:10.1016/j.rcsop.2025.100596)
Supplement: Supplementary file 1 — Categorized feedback from patients. [file mmc1.docx]

Appendix 1: patient feedback

| **Positive Aspects** | **Barriers** | **Precursors** |
| --- | --- | --- |
| Thank you, perfect, very happy (18) | Technically not mature (2) | Controversial opinions on vitamin D (4) |
| Great campaign, very good (34) | High costs (1) | Statutory health funds should cover the costs (2) |
| Should be offered regularly/ more often, more tests like this (13) | Poor advertising (1) | How often should you get tested? (1) |
| Simple and uncomplicated (5) | Long waiting time (1) | Too expensive (1) |
| Competent counselling, very friendly (6) | Not convinced by the measuring technology (1) | Clinical implications for levels out of range (1) |
| Useful for prevention (6) |  |  |
