# Supplementary material for: Vitamin D testing in pharmacies: Results of a federal screening campaign
Source: Explor Res Clin Soc Pharm. 2025 Mar 25;18:100596. doi: 10.1016/j.rcsop.2025.100596 (PMC11997348; doi:10.1016/j.rcsop.2025.100596)
Supplement: Supplementary file 2 — Categorized feedback from pharmacists. [file mmc2.docx]

Appendix 2: pharmacist feedback

| **Positive Aspects** | **Barriers** | **Precursors** |
| --- | --- | --- |
| Great idea and campaign (5) | Late/too late delivery of the device (10) | Earlier training for the device (3) |
| More campaigns like that (3) | Errors reading the test cassette (2) | Earlier delivery of the tests and the device (5) |
